# Effect of caffeine ingestion on competitive rifle shooting performance

**DOI:** 10.1371/journal.pone.0224596

**Published:** 2019-10-31

**Authors:** Håvard Nygaard, Steinar Riksaasen, Leif Malvin Hjelmevoll, Endre Wold

**Affiliations:** Department of health and exercise physiology, Inland Norway University of Applied Sciences, Lillehammer, Norway; Universidade Federal de Juiz de Fora, BRAZIL

## Abstract

**Purpose:**

The purpose of the present study was to test if caffeine ingestion affects rifle shooting accuracy in trained shooters.

**Methods:**

Twenty trained shooters performed 4 shooting tests in a randomized, double-blinded, placebo controlled crossover design; 2 identical tests after placebo ingestion and 2 after ingestion of 300 mg caffeine. The tests consisted of 30 shots in prone position and 30 in standing position on a 10 ring electronic target, on a distance of 50 metres, without any time limit, at rest.

**Results:**

Caffeine supplementation entailed a mean decrease in shooting performance by 11.8 points (95% CI: 6.7 to 17.0, effect size: 0.9). This was primarily a result of an 11.3 (95% CI: 7.2 to 15.4, effect size: 0.9) point decrease during shooting in standing position and not in prone position (0.6 point decrease, 95% CI: -2.1 to 3.2, effect size: 0.1).

**Conclusions:**

We conclude that prior ingestion of 300 mg caffeine impairs rifle shooting accuracy in trained shooters when performed in standing but not in prone position.

## Introduction

Caffeine is the most used psychoactive stimulant in the world, occurring naturally in coffee, tea and cacao and as an additive in beverages, such as soda and energy drinks [1]. It is widely used by athletes, probably due to its well-known positive effects on physical performance [2]. The ergogenic effect of caffeine is indicated by numerous studies on different aspects of physical performance, such as motor skills, strength, power and especially endurance performance [3–5].

Athletes, hunters and soldiers all over the world perform rifle shooting. Theoretically caffeine can either increase rifle shooting performance by increased attention, cognitive function and motor skill performance [3] or it can decrease the performance by increased heart rate [6], muscle tremor [7] and postural sway [8]. Several studies have explored the effects of caffeine on shooting performance in a combat setting, typically finding shorter target detection time [9], especially during fatigue and sleep deprivation [10–12], but not increased shooting accuracy. Nor is pistol steadiness and pistol shooting accuracy improved after caffeine ingestion [13, 14]. The effects of caffeine on rifle shooting accuracy in a noncombat setting seem to be sparsely studied. To our knowledge, the only previous study in this field was a small sample experiment including only two air rifle shooters [6]. Our purpose was therefore to test the hypothesis that caffeine ingestion affects rifle shooting accuracy in trained shooters.

## Methods

### Participants

We recruited the participants using posters and information to Norwegian biathlon teams in January 2018. We included biathletes aged 18–30 years. Twenty participants, 10 women and 10 men, 21 ± 1 years old, 69.0 ± 8.4 kg body weight, all Norwegian, completed the study. They had competed for an average of 11 ± 3 years, whereof 15 participants at national level and 5 in junior world championships in addition to national level. At time of study, seven were in the junior class and 13 in senior. They had fired an average of 7662 ± 2878 shots the year preceding enrolment, of which 66.3 ± 9.9% were fired at rest without time limit. Average weekly caffeine intake assessed by questionnaire was 613 ± 514 mg. The study was performed according to the ethical standards established by the Helsinki declaration and was approved by the local ethics committee at Inland Norway University of Applied Sciences. All participants gave their written informed consent.

### Experimental design

**Fig 1:**
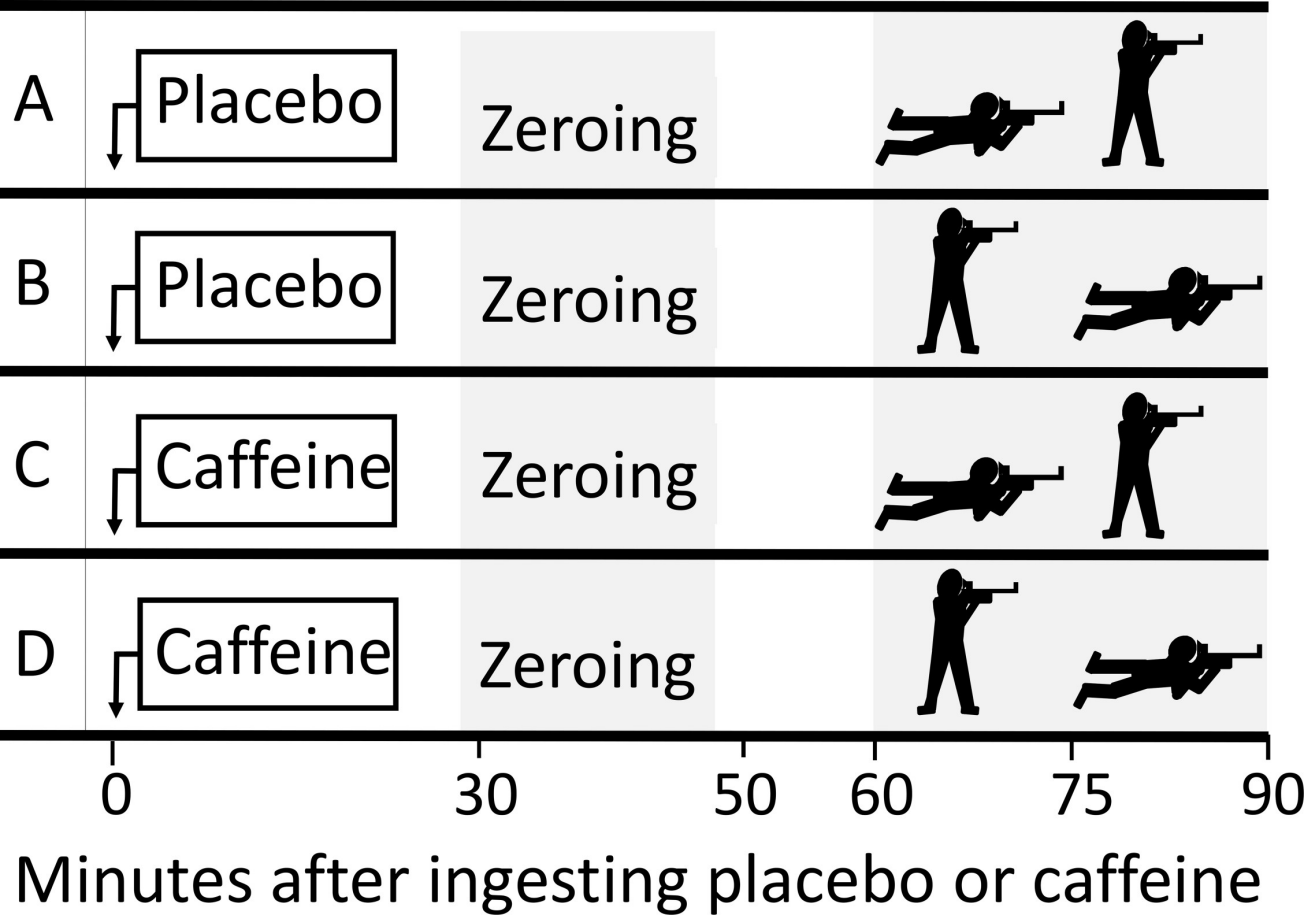
Overview of the shooting tests. In random order, each participant underwent 4 tests (A, B, C and D) on 4 different days, 2 with placebo and 2 with caffeine ingestion 60 minutes prior to start of the shooting tests. One of the tests with caffeine and one of the tests with placebo started with shooting in prone position before shooting in standing position and the other way around.

We performed the study in a randomized, double-blinded, placebo controlled crossover design. Each participant underwent 4 shooting tests on 4 different days, 2 with prior placebo and 2 with prior caffeine ingestion, Fig 1. Each test was separated by minimum 2 days and maximum 5 days. Each test for each participant was performed at the same time of the day. Order of prone and standing shooting position was randomized in a balanced way, thus one of the tests with caffeine and one of the tests with placebo started with 30 shots in prone position before 30 shots in standing position, and the other way around. Coefficient of variation for the shooting results were 2.0% for the test days with placebo and 2.5% for the days with caffeine.

### Tests

We instructed the participants not to ingest caffeine-containing food or beverages for 48 hours, and not to eat for 2 hours, prior to each test. They were also instructed to use ammunition with the same lot number on all tests, which were performed in an indoor shooting arena. The participants ingested placebo pills or pills containing 300 mg caffeine (Kragerø Tablettproduksjon AS, Kragerø, Norway) and water 60 minutes prior to start of each test, Fig 1. We registered weight with a bathroom scale (Coline B-198, Clas Ohlson AB, Sweden) and the participants reported their caffeine beverage habits in a questionnaire made by the authors, which from we calculated individual weekly caffeine intake. Intake of cola (150 mg/L), energy drinks (320 mg/L) and coffee (anticipated 100 mg caffeine per cup), were reported. The time period from 30 to 10 minutes prior to test start was used for an unlimited number of zeroing shots, and the last 10 minutes for rest and individual mental preparation, Fig 1.

The shooting test, which is developed by the Norwegian Biathlon Association, consisted of 60 shots on 50 metres on a 10 ring electronic target (maximum 10 points per shot, 10. ring diameter of 10.4 mm, Megalink AS, Vestby, Norway). During each test occasion, 60 shots were fired (30 prone + 30 standing or 30 standing + 30 prone) with the participants personal biathlon rifles (calibre 22 LR, weight of weapon ≥3.5kg and trigger pull ≥0.5 kg). To blind the participants for their own results, the monitor of the electronic scoring system was not visible during the tests. There was no time limit. After each test, we asked each participant whether he/she believed that he/she had ingested placebo or caffeine. The participants wore heart rate monitors (Polar A300, Polar Electro Oy, Kempele Finland) during the tests and a mean value from heart rates recorded 5 and 17 minutes after test start were used in analysis.

### Statistical analysis

Data were analysed with a linear mixed model in IBM SPSS statistics 24. We used subject ID as the clustering “subject” variable, scaled identity as covariance structure and we used random intercept. Test condition (control vs. caffeine) were used as a fixed factor. Residuals were checked for homogeneity. The effect of caffeine dose were calculated by including mg caffeine per kg body weight as a fixed covariate in the model. We calculated effect sizes (ES) for the effect of caffeine using Cohen’s *d*z for repeated measures [15], and interpreted the result according to Hopkins et al. [16]: *d* ≥ 0.2 = small effect, *d* ≥ 0.6 = moderate effect, *d* ≥ 1.2 = large effect.

## Results

**Fig 2:**
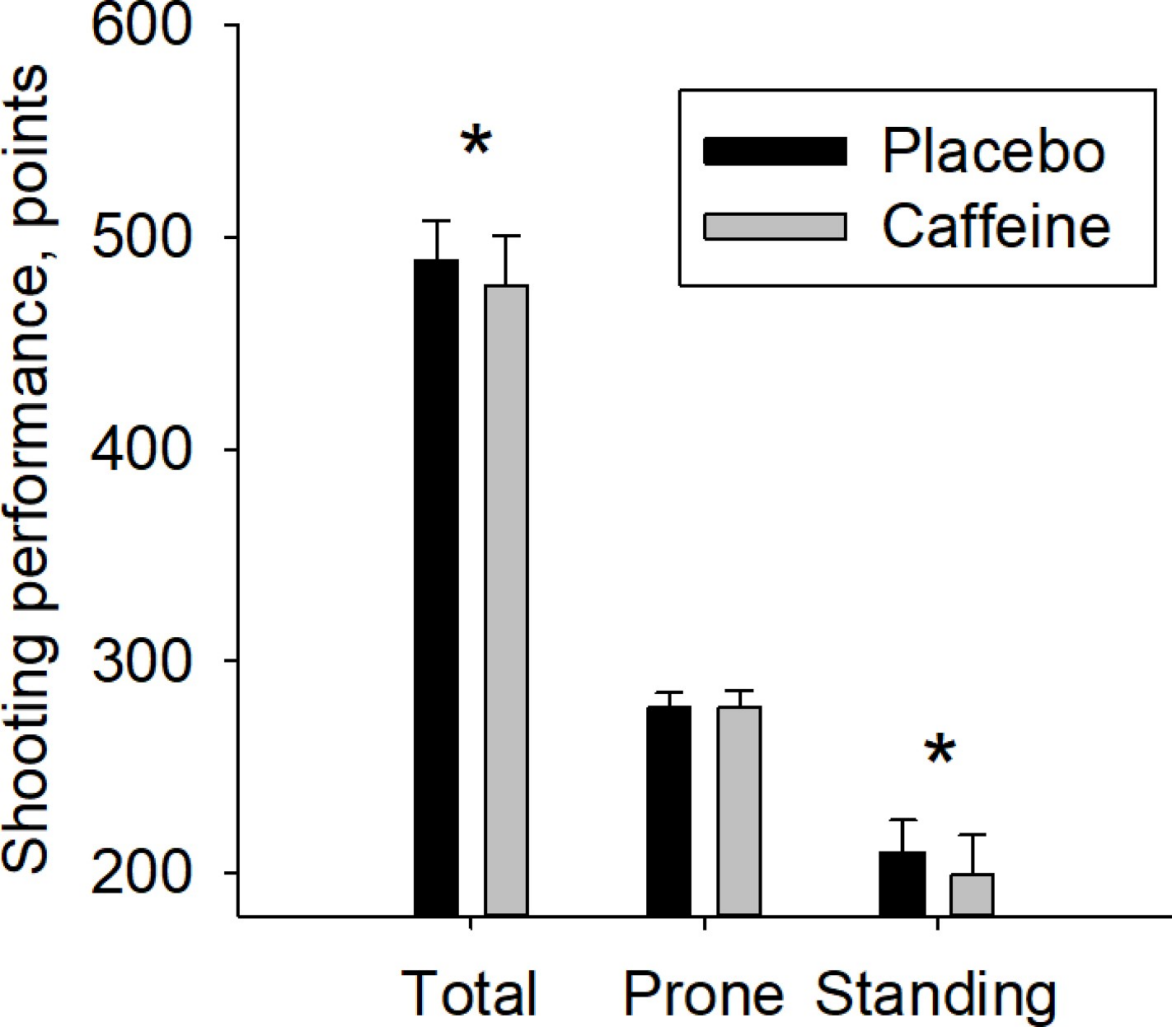
Effect of caffeine on shooting performance in function of shooting position. Total results and separate results from the shooting in prone and standing position with prior ingestion of placebo or caffeine. Mean ± standard deviation. * = p<0.001 for the difference between placebo and caffeine.

**Fig 3:**
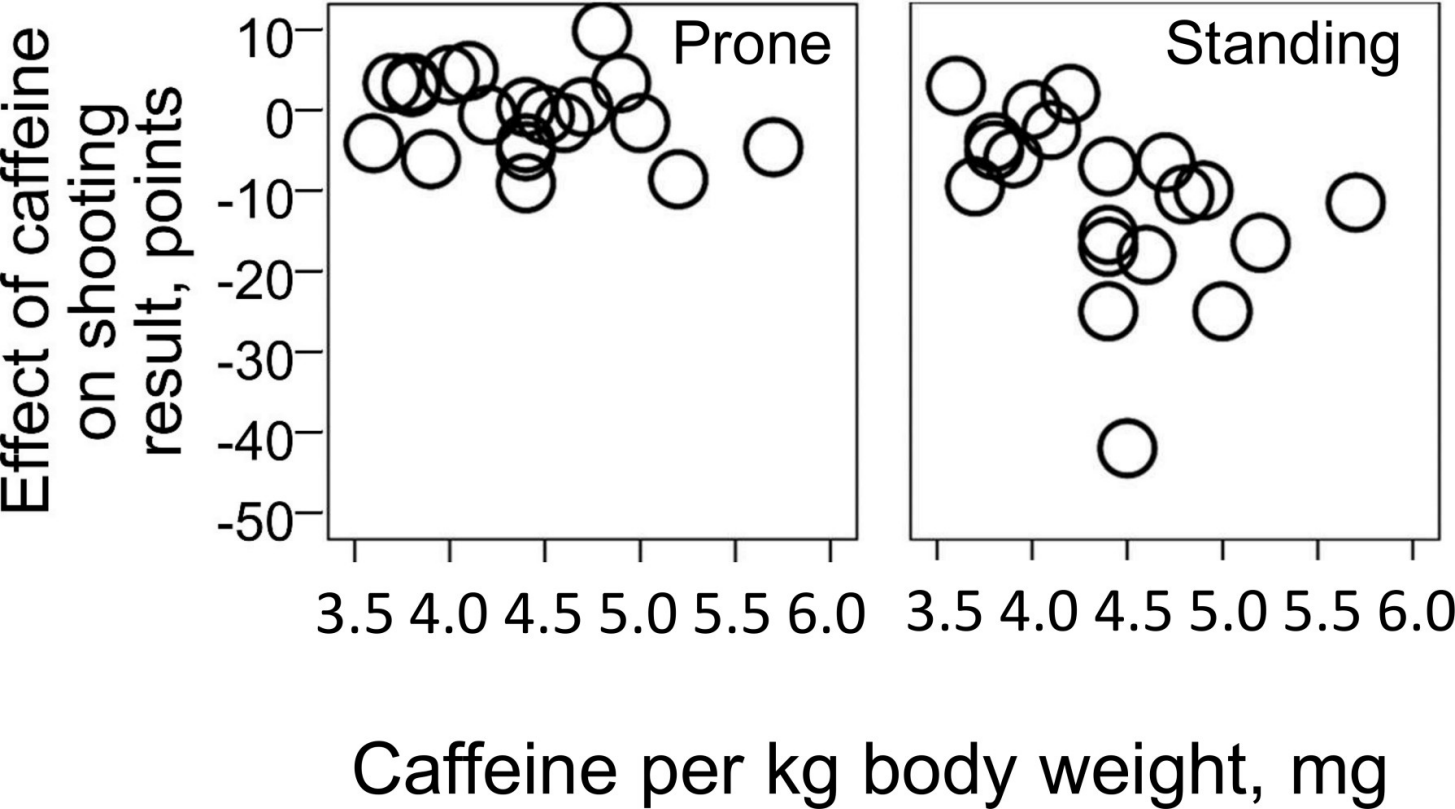
Effect of the amount of caffeine per kg bodyweight on shooting performance. Effect of the relative dose of caffeine ingested before shooting (300 mg caffeine divided by bodyweight on the x axis in both plots) on the difference between shooting results after caffeine and placebo ingestion (y axis), for the results from prone and standing position separately.

Caffeine supplementation entailed a mean decrease in shooting performance by 11.8 points (95% CI: 6.7 to 17.0). This was primarily a result of a 11.3 (95% CI: 7.2 to 15.4) point decrease during shooting in standing position, and not prone position (0.6 point decrease, 95% CI: -2.1 to 3.2), Fig 2. The effect of caffeine was 10.8 points larger (95% CI: 4.5 to 17.0) during shooting in standing position compared to prone position. Effect sizes for the effect of caffeine on shooting performance were moderate for total and standing position and negligible for prone (effect size = 0.9, 0.9 and 0.1 respectively). The amount of caffeine ingested relative to body weight ranged from 3.7 to 5.7 mg/kg. Within this range, total shooting performance decreased 10.5 points per mg ingested caffeine per kilo bodyweight (95% CI: 1.2 to 19.9) Fig 3. When adjusted for reported caffeine intake per week, the effect of the caffeine intervention on total shooting performance changed to a decrease of 15.9 points (95% CI: 7.8 to 24.0). Adjustment for correct guess on the question “whether he/she believed that he/she were on placebo of caffeine” did not affect the results, although correct answer were given in 55 out of 80 tests. Heart rate was 1 beat per minute (95% CI: -4 to 2) lower during shooting with caffeine compared to placebo. This was a result of 0 (95% CI: -5 to 4) difference in prone position and 2 beats lower per minute (95% CI: -6 to 1) with caffeine compared to placebo in the standing position. There were no significant interaction between heart rate and the effect of caffeine on shooting performance.

## Discussion

The main finding in this study was a moderate decrease in rifle shooting accuracy at rest as a result of caffeine ingestion. This effect was present during shooting in standing position, while the results from shooting in prone position were unaffected by caffeine. The strength of the study is the double-blinded randomized controlled design, which points to causality between the intervention and the results. Furthermore, causality is supported by the observed dose-response relationship between caffeine dose and the negative effect on performance in standing position and lack thereof in prone position. Although shooting techniques and context are diversified, previous studies also seem to support the observation of no ergogenic effect of caffeine on shooting accuracy, e.g. in pistol shooting [13, 14], clay target shooting [17] and shooting in combat settings with varying degrees of fatigue and sleep deprivation [9–12]. Shooting performance seem to be improved by caffeine only when the result depend on target detection time [10–12], which is typical for a combat setting. The most comparable study to the present one is the study by Ebrahimi et al. [6] on eight shooters, in which 6 were pistol shooters and 2 were rifle shooters. They found decreased shooting performance after ingestion of 5 mg but not 3 mg caffeine per kg body weight. However, the lack of effect of 3 mg might be attributed to lack of statistical power with such a low sample size.

The present study is also limited by a relatively low number of participants, but on the other hand, caffeine is tested against placebo in duplicate, increasing the robustness of the results. An another weakness in the present study might have been that the blinding of the participants was inefficient, since a greater part answered correct on the question whether he/she had ingested placebo or caffeine on each shooting test. However, adjustment for correct answer did not affect the results. The effect of caffeine should therefore be interpreted as physiological and not the participants’ awareness of what they had consumed. The purpose of the study was not to explore the physiological mechanisms of action. However, some assumptions can be done. Since the negative effect of caffeine occurred during standing shooting only, it may be attributed to increased postural sway, which occurs after caffeine ingestion [8]. Postural sway is likely to affect the stability of hold and cleanness of triggering, two main determinants of rifle shooting performance in the standing position [18–20]. Increased muscle tremor might also be a candidate, however most previous studies have failed to show caffeine induced muscle tremor [7]. Furthermore, the decrease in shooting performance during shooting in the standing position cannot be attributed to increased heart rate, since the heart rate did not increase. The lack of effect of caffeine on heart rate is also supported by previous studies [21, 22]. Interestingly, the effect seemed to be independent of the participants habitual caffeine intake, supporting the notion that “resistance” to the ergogenic effect of caffeine is a myth [23]. However, the lack of interaction between caffeine habits and the effect of caffeine intervention might be influenced by the 48-hour withdrawal from caffeine prior to shooting tests.

The present study implicates that prior caffeine ingestion should be done with caution when shooting in standing position. The participants were biathletes. Hovewer, since we performed the study in a resting context with no time limit, we cannot conclude whether this finding can be generalized to situations involving strenuous exercise and timekeeping. Indeed, a positive effect on total biathlon performance or performance in other situations requiring physical or mental performance might still occur. With regard to endurance, there are numerous studies showing increased performance after intake of 3–6 mg/kg caffeine [5]. Previous studies have also shown that intake of >3 mg/kg caffeine can increase maximal strength and power [4], and there are some studies indicating increased motor skills, attention and cognitive function [3]. In general, the effect of caffeine on performance seem to be influenced by the degree of physical or mental fatigue in the test situation [3, 5, 24]. The results of the present study should therefore be generalized to shooting accuracy at rest only. The results seems relevant for expert shooters competing at rest also because the participants had performed the major part of their shooting training in a rested condition. The observed dose—response relationship between caffeine per kg bodyweight and shooting performance indicates that the negative influence of caffeine disappears at low doses. However, our results are limited at this point, since we used an absolute dose of caffeine. Thus, only a few of the participants ingested a caffeine dose in “the lower area of caffeine per kg bodyweight”. Normalization of caffeine dose to body mass would represent a better approach to conclude on the effect of a given dose per kg. Previous studies have also found that the positive effect of caffeine on endurance performance and attention might be preserved at relatively low doses, e.g. 200 mg or <3 mg/kg [25]. Thus, we cannot exclude that a low dose of caffeine can be optimal for rifle shooting performance or rifle shooting in combination with endurance exercise, despite our observation of impaired shooting performance after ingestion of 300 mg caffeine.

## Conclusions

Prior intake of 300 mg caffeine impairs rifle shooting accuracy at rest in trained shooters when performed in standing but not in prone position.

## Supporting information

S1 Dataset (XLSX)
